# Serpina3n/serpina3 alleviates cyclophosphamide-induced interstitial cystitis by activating the Wnt/β-catenin signal

**DOI:** 10.1007/s11255-023-03726-7

**Published:** 2023-08-18

**Authors:** Weilin Fang, Qixiang Song, Tingting Lv, Jianwei Lv, Zhikang Cai, Zhong Wang, Xin Song, Xiang Ji, Jin Huang

**Affiliations:** 1 Department of Urology and Andrology, Shanghai Pudong New Area Gongli Hospital, No. 219, Miaopu Road, Pudong New District, Shanghai, 200135 China; 2 Department of Urology, Renji Hospital, Shanghai Jiao Tong University School of Medicine, Shanghai, 201112 China

**Keywords:** Interstitial cystitis, Serpina3n/serpina3, Wnt/β-catenin, Apoptosis, Inflammation

## Abstract

**Background/objective:**

Serpina3n/Serpina3 has been identified to be implicated in inflammatory diseases, but its role in interstitial cystitis/bladder pain syndrome (IC/BPS) remains unknown. Here, we aimed to reveal serpina3n/serpina3 role in IC/BPS in vivo and in vitro.

**Methods:**

The IC/BPS model in mice was induced by intraperitoneal injection of 150 mg/kg of cyclophosphamide (CYP). HE and toluidine blue staining were used for histology assessment. Serpina3n/serpina3 expression in the bladder tissues from IC/BPS patients and mouse models were determined by qPCR, immunohistochemistry and western blotting. XAV-939 treatment was applied to inhibit β-catenin activation. Serpina3 role in modulating the growth and apoptosis of HBlEpCs, a human primary bladder epithelial cell line, was assessed by CCK-8 and flow cytometry assays.

**Results:**

Serpina3n/serpina3 expression was decreased in both human and mice bladder tissues with IC/BPS. Upregulation of serpina3n significantly alleviated CYP-induced bladder injury, with decreased mast cells and pro-inflammatory factor levels, including IL-1β, IL-6, and TNF-α, while increased IL-10 level. In addition, serpina3 overexpression inhibited the apoptosis of HBlEpCs, and increased cell growth. In mechanism, we found that serpina3 overexpression promoted the activation of wnt/β-catenin signaling. And, the inhibition of wnt/β-catenin signaling with XAV-939 abolished serpina3n/serpina3 role in protecting bladder tissues from CYP-induced cystitis, as well as inhibiting HBlEpC apoptosis.

**Conclusion:**

Serpina3n/serpina3 expression was decreased in IC/BPS. Overexpression of serpina3n could alleviate CYP-induced IC/BPS by activating the Wnt/β-catenin signal. This study may provide a new therapeutic strategy for IC/BPS.

## Introduction

Interstitial cystitis/bladder pain syndrome (IC/BPS) is a chronic and debilitating syndrome characterized by bladder filling-related pain and increased urinary frequency, urgency, and other discomforts [1]. IC/BPS is prevalent in the USA, affecting 2.7–6.5% of women and 2–4% of men [2, 3]. The pathogenesis of IC/BPS involves multiple factors, such as epithelial damage, mast-cell infiltration, pelvic floor dysfunction, and infection [4]. Inflammation is a key contributor to both human and animal IC/BPS [5, 6]. Targeting inflammation represents a promising treatment method for IC/BPS.

Human serpina3 (serpin family A member 3), previously known as alpha-1-antichymotrypsin, is a structurally conserved secretory serine protease inhibitor and is homologous to murine serpina3n [7]. Serpina3n/Serpina3 is involved in many biological processes, including complement cascade, apoptosis, proliferation, wound healing, and remodeling of the extracellular matrix by suppressing proteases [8–10]. Furthermore, there is growing evidence that Serpina3n/serpina3 plays a critical role in inflammatory responses [11–13]. Serpina3n expression can be induced by palmitic acid and leptin in N42 neurons, along with the pro-inflammatory cytokines IL-6 and TNF-α, and all three genes were down-regulated by the anti-inflammatory monounsaturated fat, oleic acid [12], suggesting that serpina3n may have a pro-inflammatory effect. However, Ho et al. [13] recently reported that systemic administration of serpina3n significantly inhibited inflammation, ameliorating colitis symptoms using single-cell transcriptomics, indicating that serpina3n has an anti-inflammatory role in mouse colitis models. However, the role of serpina3a/serpina3 in IC/BPS has not been investigated to date.

In recent years, it has been discovered that the wnt/β-catenin signaling pathway has an anti-inflammatory role and can alleviate IC/BPS. For example, Choi et al. [14] found that silencing of WNT11, WNT2b, WNT5a, and WNT10a in HBlEpCs, a primary epithelial cell line derived from the normal human bladder, resulted in fibrotic changes. Song et al. [15] demonstrated that mesenchymal stem cell therapy can alleviate IC/BPS by activating the wnt/β-catenin signaling pathway. Additionally, IPSE, a urogenital parasite-derived immunomodulatory protein, activates the Wnt/β-catenin signaling pathway to ameliorate ifosfamide-induced hemorrhagic cystitis [16]. Wnt/β-catenin agonists are promising therapeutic drugs for IC/BPS.

The objective of this study was to investigate whether serpina3n/serpina3 could activate the Wnt/β-catenin signaling pathway and attenuate IC/BPS using in vitro and in vivo experiments.

## Materials and methods

### Human bladder specimen

Bladder mucosa specimens were collected from three female IC/BPS patients (ages 45–65) via cystectomy or cystostomy. Diagnosis was based on the NIDDK IC/BPS criteria, which can be found on their website (https://www.niddk.nih.gov/health-information/urologic-diseases/interstitial-cystitis-painful-bladder-syndrome). Three additional bladder specimens were obtained from female patients who underwent radical cystectomy for bladder cancer. These patients had a single large grade-3 invasive bladder tumor, and the bladder mucosal tissue far away from the tumor was selected for study. The tissue next to the sampling point was sent for pathology, and the report indicated “normal bladder mucosa”. None of the patients received intravenous chemotherapy, bladder perfusion chemotherapy, or immunotherapy before surgery. All patients provided informed consent. This study was approved by the ethics committee of Shanghai Jiaotong University School of Medicine, Renji Hospital (No. KY2022-026-B).

### Animals

Thirty female C57BL/6 J mice (5–6 weeks old, 20–22 g; Shanghai Model Organisms) were used in this study and were randomly divided into five groups: control, model, model + lenti-NC, model + lenti-Serpina3n, and model + lenti-Serpina3n + XAV-939 groups, with six mice in each group. Mice were housed in cages (three mice per cage) with a constant humidity of 55 ± 5% and a temperature of 24 ± 1 ℃ under a 12 h light/dark cycle, with free access to standard diet and water. The animal protocols were carried out based on the ethical guidelines set out in the Guide for the Care and Use of Laboratory Animals.

### Establishment of chronic cystitis models and animal treatment

Chronic cystitis was induced by systemic intraperitoneal injection of 150 mg/kg of cyclophosphamide (CYP) for 4 consecutive days, based on a previous report [17]. CYP was dissolved in 0.9% NaCl. Control mice received 0.9% NaCl at the same time. Mice in the model + lenti-NC and model + lenti-Serpina3n groups were intravenously administered with lentiviral particles of lenti-NC and lenti-Serpina3n (5 × 107 pfu per mouse; cat no. MR206624L2V, Origene, Beijing, China) for 3 days prior to cystitis induction, respectively. The model + OE-Serpina3n + XAV-939 group received intraperitoneal injection of XAV-939 (2.5 mg/kg, Selleck Chem, Shanghai, China), an inhibitor of the Wnt/β-catenin signaling pathway, once daily for three times with an interval of 6 h of lentivirus vectors.

### Mechanical referred hyperalgesia testing

This testing was carried out 24 h after animal modeling. The lower abdominal withdrawal threshold was used to assess bladder-related pain, as previously described [18]. The up–down method with various von Frey filaments (rated at 0.4, 0.6, 1, 2, 4, 6, 8, and 15 g) was applied to measure evoked pain in the lower abdomen, the area of referred pain associated with vesical pathologies. First, the 2-g stimulus was used. Each stimulus consisted of a 6–8 s application of the von Frey filament with 5-min intervals.

### Histology

After performing mechanical hyperalgesia testing, the mice were euthanized using an overdose of isoflurane and cervical dislocation. The bladders of rats were removed through a longitudinal abdominal incision, fixed in 10% neutral-buffered formalin, dehydrated, and embedded in paraffin. The paraffin-embedded tissues were sectioned into 5-μm thickness and subjected to hematoxylin and eosin, and toluidine blue staining according to the manufacturer’s instructions.

### Enzyme-linked immunosorbent assay (ELISA)

Bladder tissues were homogenized in 0.5% Tritonx-100 normal saline, and the supernatant was collected after centrifugation. The protein concentration was detected using Biuret reagent methods by UV spectrophotometry and diluted to 2 mg/ml. The levels of TNF-α, IL-1β, IL-6, and IL-10 in the bladder homogenate were measured using ELISA technique according to the manufacturer's instructions.

### Cell culture and treatment

HBlEpCs, a primary epithelial cell line derived from normal human bladder, were maintained in Bladder Epithelial Cell Growth Medium according to the instructions. The cells were treated with XAV-939 (10 μM) for 24 h and collected for the indicated analysis.

### Immunohistochemistry

After dewaxing the tissue sections, they were immersed in citrate buffer and subjected to microwave heating for antigen retrieval. Rabbit anti-mouse serpina3 antibody (DF6185, 1:2000; Affinity Biosciences, USA) was incubated at 37 ℃ for 2 h, followed by washing five times with 0.01 M PBS (pH 7.2–7.6) for 5 min each time. The sections were then treated with a goat anti-rabbit immunohistochemistry kit [Gene Tech (Shanghai) Co., Ltd, Shanghai, China]. An appropriate dilution of the secondary antibody was added to the sections and incubated at room temperature for 30–60 min, followed by three washes with PBS for 5 min each. The staining was terminated using PBS (0.01 M) under an optical microscope (Olympus), with PBS serving as the negative control.

### Cell transfection

OE-serpina3 (overexpressing plasmid of serpina3; cat no. RC200509) and sh-serpina3 (shRNAs used to downregulate serpina3; cat no. TL309540V) in HBlEpC were transiently transfected with Lipofectamine 2000 and polybrene, respectively.

### CCK-8 assay

Cell proliferation was tested using the Cell Counting Kit-8 (CCK-8, Beyotime, Beijing, China). Cells were seeded in 96-well plates with 5000 of each well. After 1, 2, 3, 4, and 5 days of cell transfection, cells were incubated with CCK-8 solution for 4 h at 37 ℃. OD values at 450 nm were measured by a SpectraMax M2e (Molecular Devices, San Jose, CA, USA).

### Flow cytometry assay

The cells were harvested and resuspended in 1 × Binding Buffer to a density of 1 × 10^6^/mL. Then, the cells were fixed with 2 mL methanol for 30 min at 4 ℃, followed by apoptosis detection through incubation with Annexin V and propidium iodide (PI) solution (BestBio, Shanghai, China). Subsequently, the staining cells were resuspended in 500 μL binding buffer and analyzed by flow cytometry (BD Bioscience, USA). Flowjo 7.6 software was used for apoptosis analysis.

### Quantitative reverse transcription-PCR (qPCR)

Total RNA extraction was carried out by TRIzol reagent (Invitrogen, USA), and then, the RNA was subjected to cDNA synthesis with the help of PrimeScript RT Master Mix kit (RR036A; Takara). Next, the cDNA was applied to PCRs detection with 2 × SYBR Green PCR Mastermix (Solarbio, Beijing, China) in e 7500 Real-Time PCR System (Applied Biosystems, USA). Primers are listed in Table 1.

**Table 1:** Primer sequences

Gene	Forward (5′–3′)	Reverse (5′–3′)
Mus-serpina3n	CAACCCTGAACATCGGGAGT	TGCAGTCTACAGAGCTGAAACC
Wnt2b	GTGTAGACACGTCCTGGTGG	TGTCTGGGTAGCGTTGACAC
Wnt5a	TTGTTGCTCCGGCCCAGAA	CCGGAACTGGTACTGGCATT
Wnt8a	GCCTATCTGACCTACACCGC	GCTCTGGCATCCTTCCCTTT
Wnt10a	TTGACATTCCTCCGCTCACC	TAGTTTTCTTCCCCGGTGCC
Wnt11	AAGAAGCTATCCTCGCCGC	GGATAGGGAGAGTGCGGAAC
β-catenin	GTCAGTGCAGGAGGCCG	GACCCTCTGAGCCCTAGTCA
Homo-Serpina3	CCTGAGGCAGAGTTGAGAATG	TCTTGGCATCCTCCGTGAAC
Wnt5a	CTCCTCTCGCCCATGGAAT	TGCAGTTCCACCTTCGATGT
Wnt8a	AGTGCCTACAGAACAGCCAC	TTGGACATGGTCACATGCCT
Wnt10a	GGTGCTCCTGTTCTTCCTACTG	GCATCCAGTTGTAAGCGGTG
Wnt11	TCAGAATGTTCTGCGGGACC	CTGCCGAGTTCACTTGACGA
β-catenin	CTGAGGAGCAGCTTCAGTCC	GGCCATGTCCAACTCCATCA

### Western blotting assay

Total protein extraction was carried out using lysis buffer (Solarbio, Beijing, China) with 1% protease inhibitor (Solarbio). After centrifugation at 4 ℃ for 30 min, Bicinchoninic acid Protein Assay kits (Thermo Fisher Scientific) were applied to examine protein concentrations in the light of specifications. Then, the proteins were added to the 10% SDS-polyacrylamide gel and submitted to electrophoresis, and then transformation to the polyvinylidene difluoride membranes (PVDF; Millipore, Billerica, MA, USA). Subsequently, the membranes were probed with primary antibodies overnight at 4 ℃ after being blocked in 5% non-fat milk for 1 h at room temperature. Anti-β-actin antibody (1:5000 dilution; cat no. ab8226, Abcam), anti-serpina3n antibody (1:1000 dilution, cat no. AF4709, R&D Systems, Inc., MN, USA), anti-serpina3 (1:2000 dilution, cat no. ab180492, Abcam), anti-Bcl-2 antibody (1:1000 dilution, cat no. ab32124, Abcam), anti-Bax antibody (1:2000 dilution, cat no. ab32503, Abcam), anti-cleaved caspase-3 (1:500 dilution, cat no. ab2302, Abcam), anti- phosphorylation (p)-β-catenin (1:500 dilution, cat no. ab75777, Abcam), and anti-β-catenin (1:5000 dilution, cat no. ab32572, Abcam) were used in this study. Next day, the membranes were incubated with the HRP-conjugated secondary antibodies at room temperature for 1 h. Protein signaling was measured using the ProfiBlot-48 (Tecan, Switzerland) with the help of ECL reagent (Millipore, USA) and quantified using ImageJ software.

### Statistical analysis

Data of this study are expressed as the mean ± standard deviation (SD). The statistical analysis was performed using SPSS 23.0. Comparisons between two and three groups were carried out with Student’s *t* test and one-way ANOVA followed by the Bonferroni test, respectively. *P* < 0.05 is considered statistically significant.

## Results

### Serpina3/serpina3n expression was decreased in IC/BPS

**Fig. 1:**
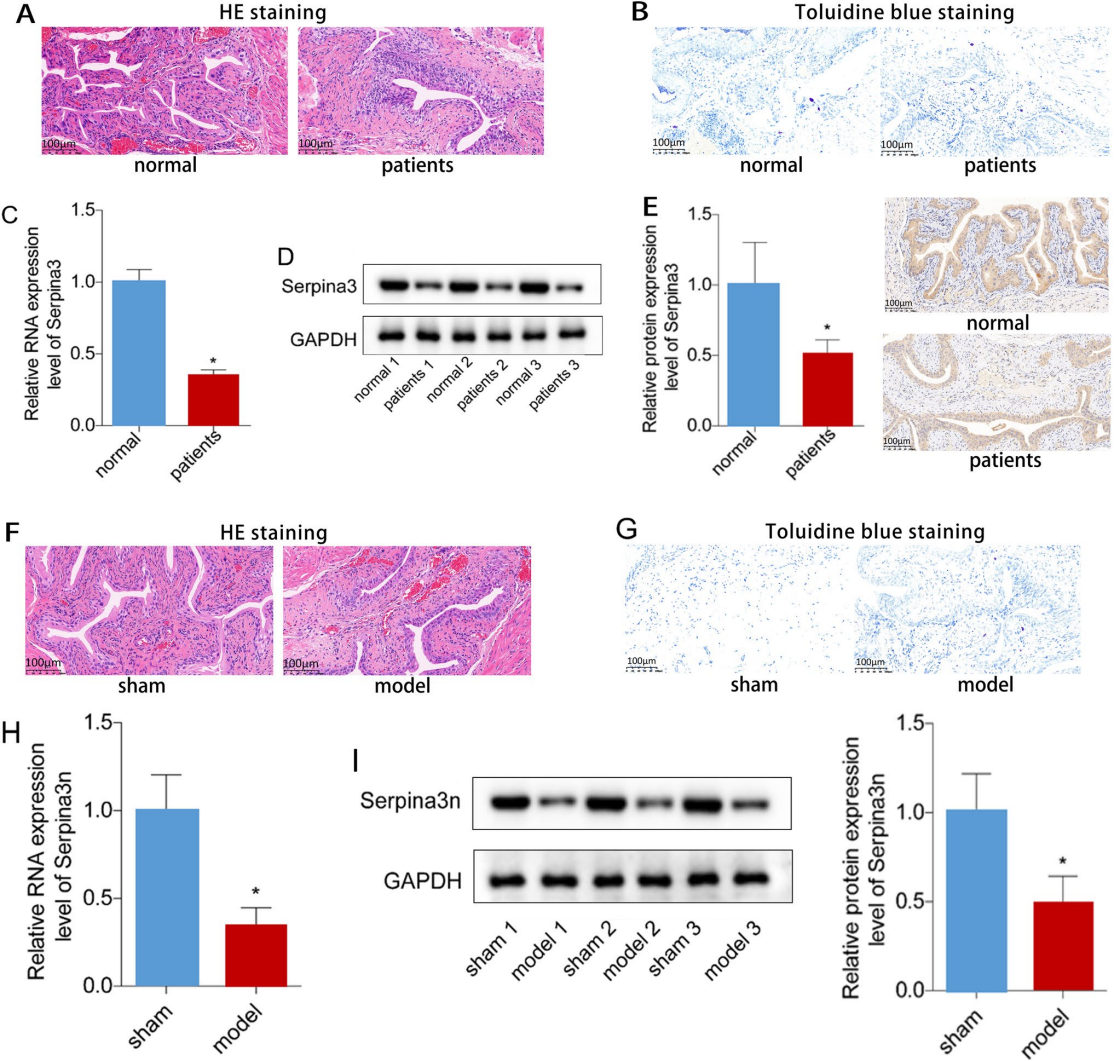
Serpina3/serpina3n expression was down-regulated in IC/BPS. **A** Assessment of the pathological changes in human bladder tissues by HE staining (× 200). **B** Evaluation of mast-cell infiltration in human bladder tissues using toluidine blue staining (× 200). **C**, **D** The mRNA and protein levels of serpina3 in the bladder tissues from IC/BPS patients and healthy individuals were tested by qPCR and western blotting. **E** Assessment of the pathological changes in mouse bladder tissues by HE staining (× 200). **F** Evaluation of mast-cell infiltration in mouse bladder tissues using toluidine blue staining (× 200). **G**, **H** The mRNA and protein levels of serpina3 in the bladder tissues from IC/BPS patients and healthy individuals were tested by qPCR and western blotting. (*P < 0.05)

To investigate the role of serpina3/serpina3n in IC, we examined its expression levels in bladder mucosa tissues from IC/BPS patients and mouse models. Compared to control samples, bladder mucosa was thinner and edema was observed in the IC/BPS group (Fig. 1A). Toluidine blue staining revealed that the number of mast cells in the bladder mucosa of the IC/BPS group was significantly increased compared to the control group (Fig. 1B). We also found that serpina3 expression was decreased in the bladder tissues of IC/BPS patients (Fig. 1C, D). We further used immunohistochemistry to label serpina3 in the bladder tissue of IC/BPS patients, discovering the expression of serpina3 in the urothelial cells of both normal individuals and IC/BPS patients. This concurrently validated the trend of down-regulated serpina3 presentation in bladder tissue, as shown in both mRNA and protein detections (Fig. 1E). To confirm these results, we induced IC/BPS in mice using CYP. The bladder mucosa of the model group showed obvious epithelial injury, inflammatory cell infiltration, and edema in the mucus and lamina propria compared to the control group (Fig. 1F). Serpina3n expression in mouse IC/BPS models was lower than that of the control group (Fig. 1H, I). These findings indicate that serpina3/serpina3n expression is decreased in IC/BPS.

### Serpina3n overexpression alleviates IC/BPS in mouse models

**Fig. 2:**
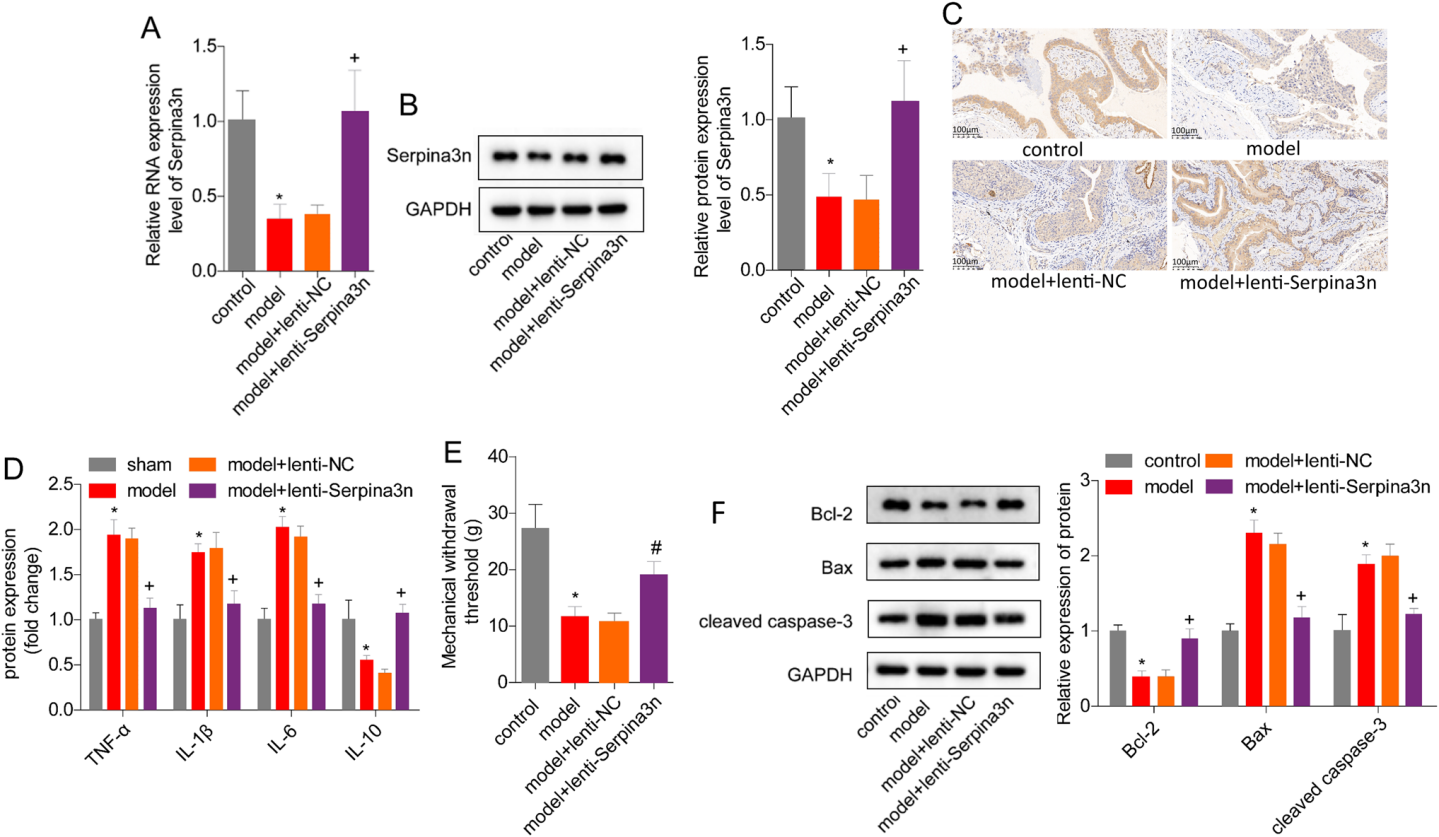
Serpina3n assuaged mouse IC/BPS induced by CYP. **A**, **B** The mRNA and protein levels of serpina3 in the bladder tissues of mice from control, model, model + lenti-NC, and model + lenti-serpina3n groups. **C** ELISA was used to detect the expression of IL-1β, IL-6, TNF-α, and IL-10 in mouse bladder tissues. **D** The mechanical withdrawal threshold. **E** Western blotting assay was applied for the detection of apoptosis proteins, BCL-2, Bax, and cleaved caspase-3. (*P < 0.05, vs. control group; + *P* < 0.05, vs. model + lenti-NC group)

To investigate the role of serpina3n in the progression of IC/BPS, we administered lenti-serpina3n intravenously in mice with CYP-induced IC/BPS (model + lenti-serpina3n group) and compared the results with a control group (model + lenti-NC group). We found that serpina3n expression was significantly increased in the bladder tissues of the model + lenti-serpina3n group compared to the control group (Fig. 2A, B). Upon the quantitative analysis of protein and gene expression, we employed immunohistochemistry to detect the expression of serpina3n in mouse bladder tissue. The model group showed a significant increase in urothelial serpina3n expression (Fig. 2C). Compared to the control group (model + lenti-NC group), the urothelial expression of serpina3n in the model + lenti-serpina3n group was significantly increased (Fig. 2C), which is consistent with the results of protein and gene quantitative analyses. We also evaluated the effect of serpina3n on inflammatory factors in bladder tissues and observed that serpina3n upregulation resulted in decreased levels of TNF-α, IL-1β, and IL-6, and increased levels of IL-10 in the model + lenti-serpina3n group as compared to the model + lenti-NC group (Fig. 2D). Moreover, we observed that serpina3n overexpression significantly reversed the decrease in mechanical withdrawal threshold in the cystitis models (Fig. 2E), as well as the decrease in expression levels of Bcl-2 and the increase in expressions of Bax and cleaved caspase-3 (Fig. 2F). These results suggest that serpina3n overexpression can alleviate CYP-induced bladder injury, inflammation, and cell apoptosis in IC/BPS.

### Serpina3 inhibits normal bladder cell apoptosis through activating of the wnt/β-catenin signaling

**Fig. 3:**
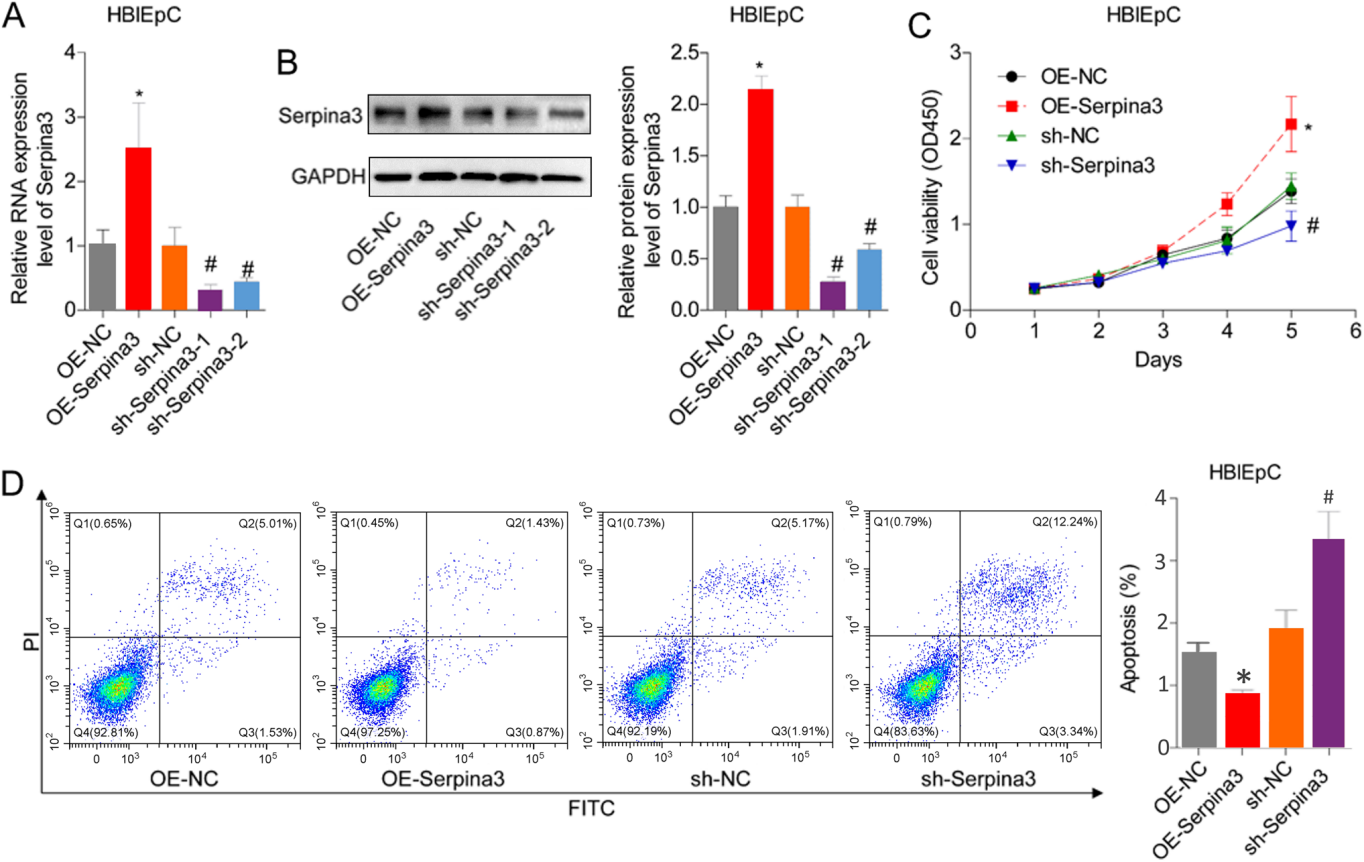
Serpina3 promoted the growth of bladder cells. **A**, **B** The mRNA and protein levels of serpina3 were detected by qPCR and western blotting in HBlEpCs transfected with sh-NC, sh-serpina3-1, sh-serpina3-2, OE-NC, and OE-serpina3. **C**, **D** Serpina3 effect on cell growth and apoptosis was detected by CCK-8 and flow cytometry assay in HBlEpCs transfected with sh-NC, sh-serpina3, OE-NC, and OE-serpina3. (*P < 0.05, vs. OE-NC group; #*P* < 0.05, vs. sh-NC group)

We further investigated the role of serpina3 in bladder cell apoptosis and proliferation using gain-of-function and loss-of-function experiments in vitro. Transfection with OE-serpina3 significantly increased serpina3 expression, while sh-serpina3 decreased serpina3 expression in HBlEpCs (Fig. 3A, B). CCK-8 and flow cytometry assays revealed that serpina3 overexpression promoted cell proliferation (Fig. 3C) and decreased cell apoptosis (Fig. 3D), while depletion of serpina3 had the opposite effect.

**Fig. 4:**
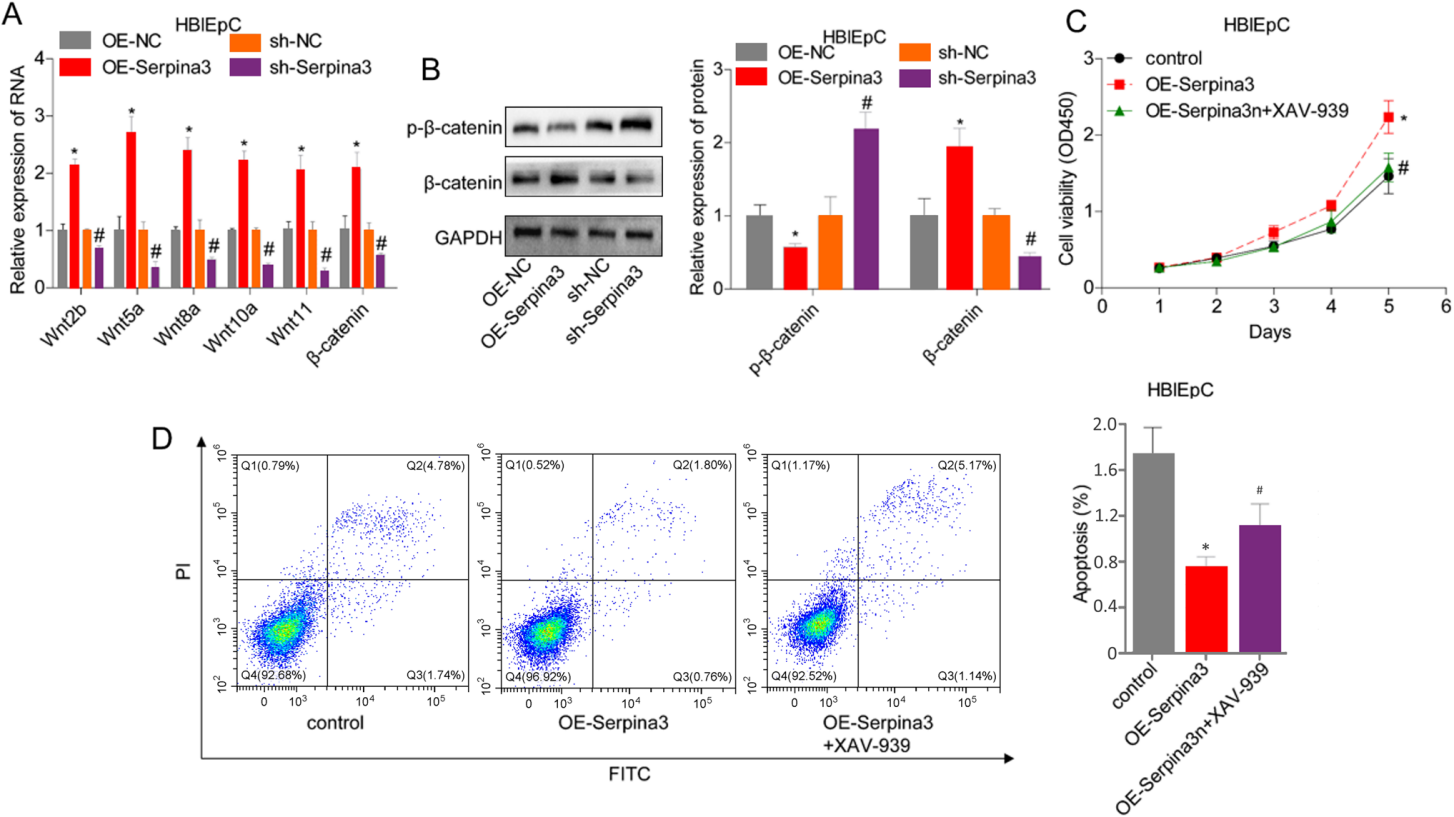
XAV-939 abolished serpina3 role in modulating HB1EPCs growth and apoptosis. **A** QPCR was applied to detect the mRNA levels of wnt2b, wnt5a, wnt8a, wnt10a, wnt11, and β-catenin in HBlEpCs transfected with sh-NC, sh-serpina3, OE-NC, and OE-serpina3. **B** The expressions of p-β-catenin and β-catenin in HBlEpCs transfected with sh-NC, sh-serpina3, OE-NC, and OE-serpina3 were detected using western blotting (*P < 0.05, vs. OE-NC group; #*P* < 0.05, vs. sh-NC group). Cell growth and apoptosis was detected by CCK-8 (**C**) and flow cytometry assay (**D**) in HBlEpCs treated with OE-NC, OE-serpina3, and OE-serpina3 + XAV-939 (*P < 0.05, vs. OE-NC group; #*P* < 0.05, vs. OE-serpina3 group)

We then explored the involvement of the wnt/β-catenin signaling pathway in serpina3-mediated inhibition of bladder cell apoptosis. Compared to the OE-NC group, serpina3 overexpression led to significant increases in the mRNA levels of wnt2b, wnt5a, wnt8a, wnt10a, wnt11, and β-catenin (Fig. 4A), as well as the protein expression levels of β-catenin and p-β-catenin (Fig. 4B). To further confirm the role of the wnt/β-catenin signaling pathway, we conducted rescuing experiments using XAV-939, a selective inhibitor of this pathway. The results showed that the increase in cell proliferation and decrease in cell apoptosis induced by serpina3 overexpression were abolished when the cells were treated with XAV-939 (Fig. 4C, D). These findings suggest that serpina3 overexpression inhibits normal bladder cell apoptosis and promotes cell proliferation through the activation of the wnt/β-catenin signaling pathway.

### Serpina3n overexpression alleviates IC/BPS through activating of the wnt/β-catenin signaling in mouse models

**Fig. 5:**
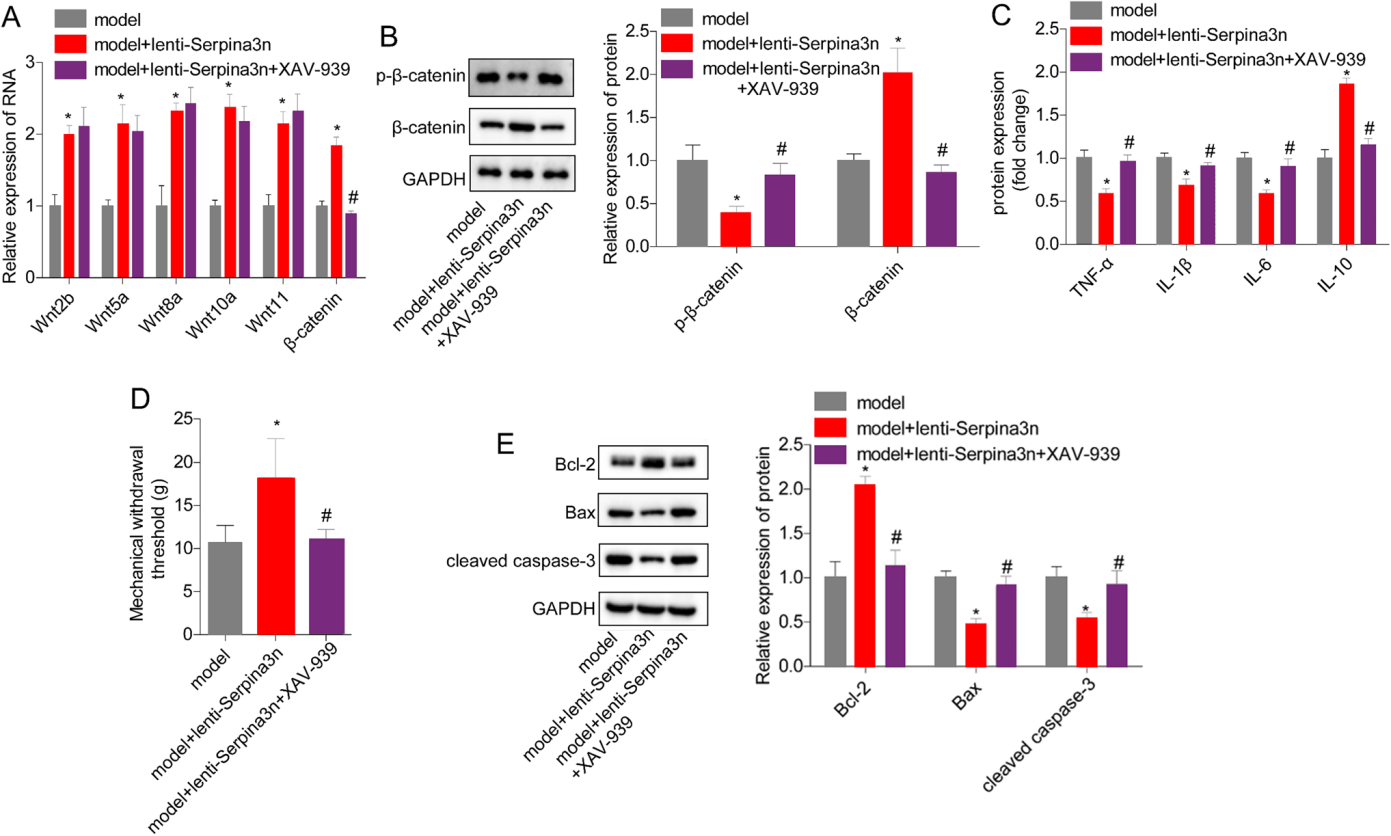
Serpina3n overexpression alleviated IC/BPS through activating of the wnt/β-catenin signaling in mouse models. **A** QPCR was applied to detect the mRNA levels of wnt2b, wnt5a, wnt8a, wnt10a, wnt11, and β-catenin in mouse bladder tissues of different groups. **B** The expression of p-β-catenin and β-catenin in mouse bladder tissues. **C** ELISA was used to detect the expression of IL-1β, IL-6, TNF-α, and IL-10 in mouse bladder tissues. **D** The mechanical withdrawal threshold. **E** Western blotting assay was applied for the detection of apoptosis proteins, BCL-2, Bax, and cleaved caspase-3. (*P < 0.05, vs. model group; #*P* < 0.05, vs. model + lenti-serpina3n group)

Furthermore, we investigated the role of the wnt/β-catenin signaling pathway in serpina3n-induced alleviation of IC/BPS in vivo. We found that the mRNA levels of wnt2b, wnt5a, wnt8a, wnt10a, wnt11, and β-catenin, as well as the protein expression levels of β-catenin and p-β-catenin, were significantly increased in the model + lenti-serpina3n group compared to the model group. However, when treated with XAV-939, a selective inhibitor of the wnt/β-catenin signaling pathway, these effects were neutralized (Fig. 5A, B). In addition, XAV-939 treatment increased the levels of TNF-α, IL-1β, IL-6, and IL-8, and decreased the level of IL-10 in the bladder tissues, as well as decreased the mechanical withdrawal threshold (Fig. 5C, D). Furthermore, XAV-939 treatment rescued the increase in the expression of Bcl-2 and the decreases in the expression of Bax and cleaved caspase-3 caused by serpina3n overexpression (Fig. 5E). These findings suggested that serpina3n overexpression alleviated IC/BPS through activating the wnt/β-catenin signaling pathway in vivo.

## Discussion

This study aimed to investigate the potential role of serpina3/serpina3n in the progression of IC/BPS, both in vivo and in vitro. Our findings, for the first time, suggest that overexpression of serpina3/serpina3n could provide protection against CYP-induced IC/BPS and enhance normal bladder cell growth.

Serpina3 is a significant member of the serine peptidase inhibitors, and was previously considered an acute-phase protein, because its synthesis increases when inflammation occurs [19]. However, later studies have shown inconsistent and even contradictory results. By inhibiting cathepsin G, a pro-inflammatory enzyme released at inflammation sites that contributes to the activation of inflammatory cytokines, serpina3 should inhibit inflammation and apoptosis [20, 21]. For example, linagliptin has been reported to effectively inhibit myocardial inflammation by increasing serpina3n activity and repressing cathepsin G activity in mice with experimental autoimmune myocarditis [22], suggesting that serpina3n exerts an anti-inflammatory role. Additionally, systemic administration of serpina3n has been shown to significantly suppress inflammation and subsequently improve colitis [13]. Since interstitial cystitis/bladder pain syndrome (IC/BPS) is an inflammatory disease [23], and the method of IC/BPS modeling through cyclophosphamide (CYP) has been verified in many studies [24, 25], we investigated whether serpina3/serpina3n plays a role in the progression of CYP-induced IC/BPS. Initially, we found that serpina3/serpina3n expression was decreased in the bladder tissues of patients with IC/BPS and mouse models induced by CYP. Subsequently, we explored the role of serpina3n in the progression of CYP-induced IC/BPS. We observed that serpina3n overexpression significantly reduced the epithelial injury, inflammatory cell infiltration, and edema in the mucus and lamina propria of IC/BPS mice, as well as decreased the mast-cell number and increased the mechanical withdrawal threshold. The increase and activation of mast cells are considered as one of the specific pathological changes of IC/BPS [26, 27], and it has also been found that serpina3/serpina3n expression is negatively correlated with mast-cell activation [28]. These results indicated that serpina3n can alleviate CYP-induced IC/BPS. Additionally, we observed serpina3n underexpression in the chronic IC/BPS models induced by CYP administration. The results showed that serpina3n overexpression inhibited the production of pro-inflammatory factors, including TNF-α, IL-1β, and IL-6, while increasing the level of IL-10, an anti-inflammatory factor. This indicates that serpina3 can mitigate the inflammatory reactions induced by IC/BPS.

In addition to inflammation, increased urothelial apoptosis is also closely associated with IC/BPS [29]. Therefore, we investigated the role of serpina3 in modulating apoptosis and proliferation in human normal bladder cells. Our results showed that overexpression of serpina3n significantly inhibited the expression of apoptosis markers, including cleaved caspase-3 and Bax, while increasing the anti-apoptotic marker Bcl-2 in the bladder tissues of IC/BPS mice. These findings suggest that serpina3n can inhibit urothelial apoptosis, which was further confirmed by in vitro experiments showing that upregulation of serpina3 increased the proliferation and inhibited the apoptosis of human normal bladder cells (HBlEpCs). This anti-apoptotic role of serpina3/serpina3n in IC/BPS is consistent with its previously identified roles in cancers [30, 31].

In terms of mechanism, we found that serpina3/serpina3n can activate the wnt/β-catenin signaling pathway. Another member of the serine proteinase inhibitor family, serpina3k, has been shown to exert anti-inflammatory and anti-angiogenic effects by blocking Wnt signaling [32]. In IC/BPS cells, the activity of β-catenin is inhibited due to its phosphorylation at ser33,37/thr41 [33, 34], suggesting that β-catenin activity is inhibited in IC/BPS cells. Activation of the wnt/β-catenin pathway has been shown to ameliorate cystitis through mesenchymal stem cell therapy [15] and IPSE, a urogenital parasite-derived immunomodulatory protein [16], indicating that the wnt/β-catenin pathway may be a promising target for IC/BPS treatment. Interestingly, we found that serpina3 can induce the activation of β-catenin. Yuan et al. [28] demonstrated that serpina3 is mainly enriched in several signaling pathways, including MAPK, TNF, P53, PI3K–Akt, and nuclear factor-κB. In addition, serpina3 has been shown to act as a transcriptional activator of PI3Kδ expression in HCC (hepatocellular carcinoma) progression, promoting HCC proliferation [35]. Since PI3K/Akt can regulate β-catenin signaling [36], we speculate that the activation of β-catenin signaling by serpina3/serpina3n may be due to PI3K/AKT signaling. We intend to further explore this in future studies.

In this study, we demonstrated that overexpression of serpina3/serpina3n alleviated IC/BPS through the activation of the Wnt/β-catenin signaling pathway, which was further confirmed by the significant reversal of serpina3n-mediated mitigation of IC/BPS by XAV-939, a β-catenin signaling inhibitor, in vivo, as well as impaired serpina3’s effect on inhibiting bladder epithelial cell apoptosis in vitro. These results suggested that serpina3n/serpina3 inhibited the progression of IC/BPS through activation of the β-catenin signaling pathway.

However, we acknowledge that the Wnt family members examined in this study can also activate non-canonical (β-catenin-independent) Wnt pathways, which we did not investigate. Although we did not explore the role of non-canonical Wnt signaling in IC/BPS, we agree that this is an interesting area for future study. Thus, our findings provide a basis for further exploration of the role of Wnt signaling in IC/BPS, including both canonical and non-canonical pathways, which may have important implications for the development of novel therapeutic strategies.

In addition, we recognize that the sample size of patients with IC/BPS in our study was relatively small, which may limit the generalizability of our findings. Therefore, future studies with larger sample sizes are needed to further confirm our results. Another limitation is that we used intravenous injection to overexpress serpina3n, which cannot exclude the possibility of its effects on other tissues or organs during the injection period. However, we used mechanical referred hyperalgesia testing to assess bladder-related pain, which is only associated with bladder pathology. Furthermore, we did not examine the effects of our treatments on cystometry, an important evaluation of bladder overactivity in IC/BPS. Future studies should evaluate the effect of serpina3n overexpression on both bladder-related pain and bladder overactivity in larger patient populations.

## Conclusion

In this study, we found that the expression of serpina3n/serpina3 was decreased in IC/BPS. By overexpressing serpina3n, we observed an alleviation of CYP-induced apoptosis, inflammation, and pain, leading to a mitigation of IC/BPS through the activation of the Wnt/β-catenin signaling pathway. These results may provide a new therapeutic strategy for IC/BPS.
